# Transient pentameric IgM fulfill biological function—Effect of expression host and transfection on IgM properties

**DOI:** 10.1371/journal.pone.0229992

**Published:** 2020-03-12

**Authors:** Julia Hennicke, Linda Schwaigerlehner, Clemens Grünwald-Gruber, Isabelle Bally, Wai Li Ling, Nicole Thielens, Jean-Baptiste Reiser, Renate Kunert

**Affiliations:** 1 Department of Biotechnology, University of Natural Resources and Life Sciences, Vienna, Austria; 2 Department of Chemistry, University of Natural Resources and Life Sciences, Vienna, Austria; 3 Institut de Biologie Structurale, UMR 5075, Université Grenoble Alpes, CEA, CNRS, IBS, Grenoble, France; Cleveland Clinic, UNITED STATES

## Abstract

Recombinant production of IgM antibodies poses a special challenge due to the complex structure of the proteins and their not yet fully elucidated interactions with the immune effector proteins, especially the complement system. In this study, we present transient expression of IgM antibodies (IgM617, IgM012 and IgM012_GL) in HEK cells and compared it to the well-established stable expression system in CHO cells. The presented workflow investigates quality attributes including productivity, polymer distribution, glycosylation, antibody structure and activation of the classical complement pathway. The HEK293E transient expression system is able to generate comparable amounts and polymer distribution as IgM stably produced in CHO. Although the glycan profile generated by HEK293E cells contained a lower degree of sialylation and a higher portion of oligomannose structures, the potency to activate the complement cascade was maintained. Electron microscopy also confirmed the structural integrity of IgM pentamers produced in HEK293E cells, since the conventional star-shaped structure is observed. From our studies, we conclude that the transient expression system provides an attractive alternative for rapid, efficient and high-throughput production of complex IgM antibodies with slightly altered post-translational modifications, but comparable structure and function.

## Introduction

Immunoglobulins of subclass M (IgM) are considered as the main actors in the protection against humoral microbial infection and in the mediation of cell debris clearance by activating and controlling the complement classical pathway and inflammation. This crucial role theoretically makes them relevant for therapeutic applications. Although clinical trials of metastatic melanoma patients with a human anti-GD2 IgM (MORAb-028) were already started, the study was terminated due to a lack of product availability (ClinicalTrials.gov Identifier: NCT01123304, update: July 2019). Production of IgMs is a cost-intensive and tedious process no matter if isolated from serum or recombinantly expressed in stable cell lines [1–3]. The challenges in recombinant IgM production are an adequate productivity, homogenous polymerization, purity, structural integrity and biological function. Even though pentamers and hexamers are the predominant forms of IgM in human serum, aggregated or incomplete polymers were described for recombinantly produced IgMs resulting in reduced product quality [2,4–6]. Another important quality attribute is the glycosylation since it may affect IgM secretion, cytolytic activity, immunogenicity or pharmacokinetics [7–10].

In the herein presented study, we evaluated if transient expression systems produce acceptable quantity, high quality and functional IgMs and thereby provide rapid, efficient and high-throughput alternatives to the established expression systems. The production of the three model antibodies IgM012, IgM012_GL and IgM617 was investigated in the human HEK293E cell line and compared to the well-established IgM producing CHO DG44 cell lines [1]. The HEK293E cell line is a frequently used system for transient expression because of its properties such as easy maintenance, robustness, ease of transfection and high yield of protein expression [11–13]. CHO DG44 cell line represents traditional and the most commonly used stable producing cell system in industrial production of recombinant protein therapeutics [14]. Our study investigates and compares the productivity of both expressions systems and several quality attributes: the polymer distribution and glycosylation patterns, as well as the in vitro functional activity to activate the classical complement pathway and their structural integrity by negative stain transmission electron microscopy (TEM).

## Materials and methods

### Genetic constructs and cell lines

The stable IgM producing CHO DG44 cell lines were generated as described in Chromikova *et al*., 2015 [1]. In short, two different pIRES vectors were used for co-transfection and random gene integration. The IgM heavy chain and dihydrofolate reductase (DHFR) genes were connected by an internal ribosome entry site (IRES) sequence and expressed under the SV40 promoter. The light chain is expressed under the cytomegalovirus (CMV) promoter and followed by the IRES and joining chain sequence. The host cell line was transfected with two pIRES constructs containing either IgM heavy chain and DHFR genes or light chain and joining chain genes (S1A Fig).

The transient IgM expression was performed in HEK293E cells with two pCEP4 constructs containing either IgM heavy chain gene or light chain and joining chain genes (S1B Fig). The pCEP4 vector encodes the *EBNA1* gene for episomal plasmid amplification and the CMV promoter upstream the gene of interest (S1B Fig). The genes encoding the IgM chains were identical to the constructs used for stable transfection. The stable expression of the adenovirus 13 S E1a in the HEK293E cells enhances transcription of CMV promoter [15].

### Production of IgM

Stable CHO DG44 cell lines and HEK293E host cell lines were seeded at 10^6^ cells/mL. Three parallel cultures of the IgM producing CHO DG44 cells were cultivated in batch mode for each model IgM. HEK293E were transfected with the constructs described in S1B Fig using polyethylenimine. 48 hours post transfection the cultures were supplemented with 0.5% tryptone N1 (TN1) and 5 mM valproic acid (VPA) to increase the protein synthesis in transient expression up to the yields of stable expression systems [16,17]. To quantify IgM titers, cell culture supernatants were analyzed with a standard μ-κ-ELISA using the respective purified IgM antibody as reference material. Antigen binding activities of all IgMs was also evaluated by ELISA and detection of IgMs deposition over coating specific antigens. As expected, all IgM preparations were able to bind their specific antigen.

### Purification

IgM antibodies were purified according to Hennicke *et al*., 2017 [18]. Briefly, POROS CaptureSelect^TM^ IgM Affinity Matrix (Thermo Fisher Scientific) was used for affinity chromatography. The IgMs were eluted with 1 M Arginine, 2 M MgCl_2_, pH 3.5. As a second purification step, the eluted IgM samples were applied on a Superose^TM^ 6 column (GE Healthcare). IgM was separated from nucleic acid contaminants with running buffer (0.1 M sodium phosphate pH 5.5, 0.2 M NaCl) at a flow rate of 0.5 mL/min.

### SDS PAGE, silver stain and immunoblotting for polymer distribution

Separation and identification of purified IgMs by SDS-PAGE and Western blot was performed as described in [1]. Silver staining was performed according to [19]. A goat anti-human μ-chain specific peroxidase antibody (1:2000, Sigma) was used to detect the μ-chain of the IgM molecule.

### Transmission electron microscopy (TEM)

60–80 ng of IgM was applied to a Mica Sheet covered with evaporated carbon film. The film was floated off in ~100 μL 2% sodium silicotungstate (SST, Agar Scientific) and fished onto a 400 mesh Cu TEM grid (Delta Microscopies). Images were taken with a Tecnai F20 TEM microscope at 200 keV.

### N-linked glycosylation

The site-specific glycan pattern was analyzed as described in [18]. Briefly, purified IgM was S-alkylated with iodoacetamide and digested with Trypsin (Promega) or with Trypsin and endoproteinase GluC (Roche). IgM fragments were separated with RP-HPLC (BioBasic C18 column, Thermo Fisher Scientific) and detected with QTOF MS (Bruker maXis 4G). MS spectra were recorded in DDA (data depended acquisition–highest peaks are selected for MS/MS fragmentation) mode in a range from 150–2200 Da. The five possible glycopeptides were identified as sets of peaks consisting of the peptide moiety and the attached N-glycan varying in the number of Gal-GlcNAc units (antennae), fucose and sialic acid residues. The theoretical masses of these glycopeptides were determined with a spreadsheet using the monoisotopic masses for amino acids and monosaccharides.

### Complement activation-ELISA

The activation of the classical complement pathway was monitored by an ELISA-assay based on detection of C4b deposition according to Bally *et al*., 2019 [20]. In brief, 200 ng of IgM were immobilised on a 96-well plate (Thermo Fisher Scientific) and incubated with 4% normal human serum (NHS), 4% C1q depleted serum (NHSΔ, CompTech) or 4% NHSΔ reconstituted with purified human C1q (4 μg/mL) [21]. NHS was obtained from the Etablissement Français du Sang Rhône-Alpes (agreement number 14–1940 regarding its use in research). Unspecific binding was prevented by saturation with 2% bovine serum albumin (BSA, Sigma Aldrich). In the course of the classical pathway activation, C4 is cleaved into C4a and C4b, which deposition was detected with a rabbit anti-human C4 polyclonal antibody (Siemens) specific for C4b and an anti-rabbit-HRP antibody conjugate (Sigma Aldrich). C4b deposition was visualized by addition of TMB (Sigma Aldrich) and a Clariostar plate reader (BMG Labtech). Screened recombinant IgM samples were produced by transient HEK293E and stable CHO DG44 and purified as described above. Polyclonal IgM isolated from human serum (Sigma Aldrich) was used as control.

## Results

### Expression and polymer distribution of IgM

**Fig 1:**
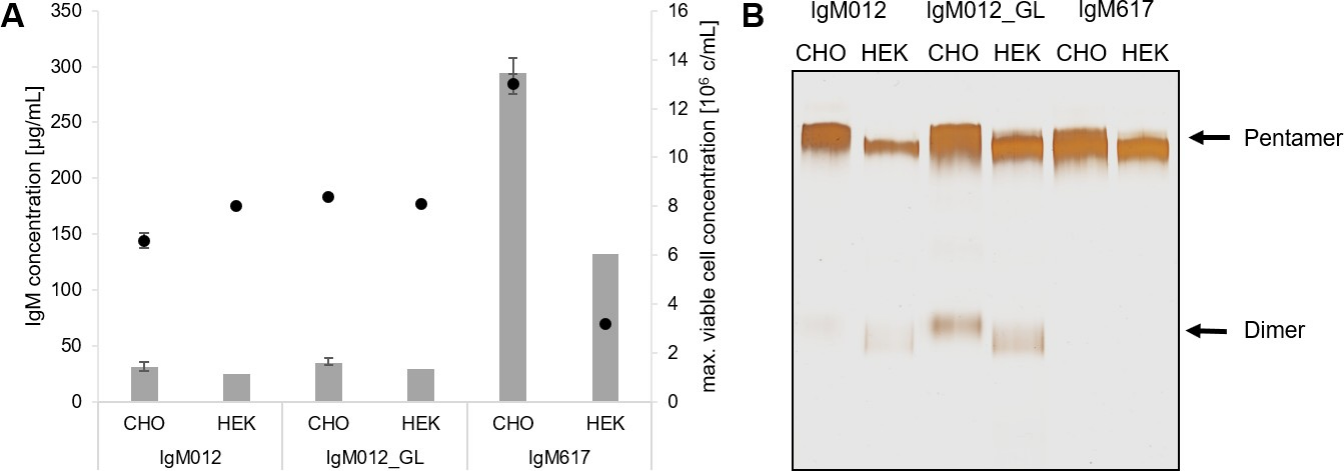
Expression and polymer distribution of IgM antibodies produced in CHO DG44 and HEK293E cells. (A) IgM concentrations are shown as bars and viable cell concentrations are represented as dots. Stable expression in CHO cells was performed in triplicates. (B) Silver staining under non-reducing conditions of purified IgM012, IgM012_GL and IgM617 produced stably in CHO cells and transiently in HEK cells. Pentameric and dimeric forms are indicated with arrows.

To assess if the transient expression is a suitable alternative to stable expression, production of IgM was investigated in both systems. Comparison of stable expression in CHO DG44 and transient expression in HEK293E showed that similar maximum viable cell concentrations result in comparable amounts of the respective IgM antibody variant (*e*.*g*., approx. 8.4 × 10^6^ cells/mL in CHO DG44 and 8.1 × 10^6^ cells/mL in HEK293E expressing 36 μg/mL and 30 μg/mL IgM012_GL, respectively) (Fig 1A), although transient expression often shows reduced product concentration compared to stable production. Also, transient transfections in HEK293E present high variabilities and only the most efficient transfections for each IgM are shown in Fig 1A. Moreover, as previously described in Chromikova *et al*., 2015 [1] for CHO production, differences in antibody HEK expression behavior were observed for all three IgM models. Indeed, similarly in both systems, production of IgM617 yields in significantly higher product titers (approx. 9-fold in CHO DG44 and 5-fold in HEK293E) compared to IgM012 and IgM012_GL [1]. To investigate the impact of the expression system on selected quality attributes of the IgMs, we evaluated the polymer formation by gel electrophoresis and glycosylation by LC-MS/MS. All IgMs were successfully produced in both cell lines as pentamers as the major IgM fraction for all model IgMs. However, purified IgM012 and IgM012_GL showed dimer formation, indicating that the differences in polymer distribution of the individual antibodies were independent of the host cell line and the expression system (Fig 1B). The presence of dimeric IgM was also observed in analytical SEC-HPLC, at least for IgM012_GL [22].

### Host-specific glycosylation of IgM

IgMs bear five potential N-glycosylation sites (GS) which can be divided into the mainly complex glycosylated GS1-3 and the primarily oligomannose type GS4 and GS5 [23]. Especially in recombinant IgM, GS1 is partially occupied also by oligomannose or hybrid glycans as well [18].

Production in HEK293E cells led to a higher mobility on the SDS gel of all protein fractions compared to production in CHO DG44 (Fig 1B and S2 Fig). This effect was especially illustrated by the dimeric portions of IgM012 and IgM012_GL, suggesting that there is a difference in the glycosylation pattern produced in the two host cell lines. As additional quality attribute, the glycosylation pattern was analyzed by LC-MS/MS. Although similar glycan patterns were found in IgMs of transiently transfected HEK293E, differences in glycosylation compared to the reference cell line CHO DG44 were observed and are summarized in Tables 1 and 2. Recombinant IgM stably produced in CHO DG44 cells comprised mainly complex type glycans, 10–16% hybrid type glycans and 20–35% oligomannose type glycans at GS1. In comparison, expression in HEK293E cells showed a minimum of ~50% oligomannose type glycans and a maximum of approximately 30% complex type glycans at GS1 (Table 1). The IgM012 produced in HEK293E had GS1 nearly completely occupied by oligomannose type indicating that the portion of less processed glycans attached to GS1 is higher in IgM produced in HEK293E cells. At GS2, the CHO DG44 cells attached predominantly complex type glycans, which were mainly sialylated while the HEK293E cells produced only 40% sialylated complex glycans, but an increased fraction of oligomannose type glycans (Table 1). This trend was even more pronounced with IgM012 produced by HEK293E. GS3 was fully occupied by complex type glycans for IgM produced in both expression systems, except for IgM012 produced in HEK293E. For our three model IgMs, transient expression in HEK293E cells resulted in a higher portion of truncated glycan structures with terminal N-acetylglucosamine, which represent less processed complex type structures.

**Table 1:** Comparison of site-specific glycosylation profile at GS1-3 of IgM produced stably in CHO cells and transiently in HEK cells. Relative abundance [%] of N-glycan types is shown.

	Glycan type	complex type			hybrid type	Oligomannose type
	model IgM	MGn/ GnGnF	galactosylated	sialylated		
Glycosylation site 1						
CHO	IgM012	1	9	43	13	35
	IgM012_GL	1	9	59	10	20
	IgM617	0	14	43	16	27
HEK	IgM012	0	1	5	1	93
	IgM012_GL	3	5	24	17	50
	IgM617	3	7	24	21	45
Glycosylation site 2						
CHO	IgM012*	47	10	32	0	1
	IgM012_GL	6	17	75	0	2
	IgM617	2	13	84	0	1
HEK	IgM012	15	2	5	0	77
	IgM012_GL	19	7	36	0	36
	IgM617	27	17	42	0	14
Glycosylation site 3						
CHO	IgM012	15	31	51	0	3
	IgM012_GL	10	21	68	0	0
	IgM617	1	18	81	0	0
HEK	IgM012	4	4	2	0	90
	IgM012_GL	39	34	27	0	0
	IgM617	37	34	29	0	0

*10% of the found glycosylation site was not glycosylated.

**Table 2:** Comparison of oligomannose structures at GS4 of IgM produced stably in CHO cells and transiently in HEK cells. Relative abundance [%] of N-glycan structures is shown.

		Man4/Man5	Man6/Man7	≥Man8
Glycosylation site 4				
CHO	IgM012	33	43	24
	IgM012_GL	24	47	29
	IgM617	21	49	30
HEK	IgM012	7	10	83
	IgM012_GL	26	32	41
	IgM617	20	39	41

Oligomannose type was the predominant glycan structure found at GS4 and GS5 of IgM produced by both expression hosts (Table 2). The number of attached mannose molecules was significantly higher in HEK293E cells compared to the oligomannose structures found in CHO DG44 cells.

All in all, glycosylation analysis revealed that transient expression in HEK293E cells leads to a less processed glycan pattern compared to stable expression in CHO DG44 cells.

### TEM of IgM variants produced by HEK293E

**Fig 2:**
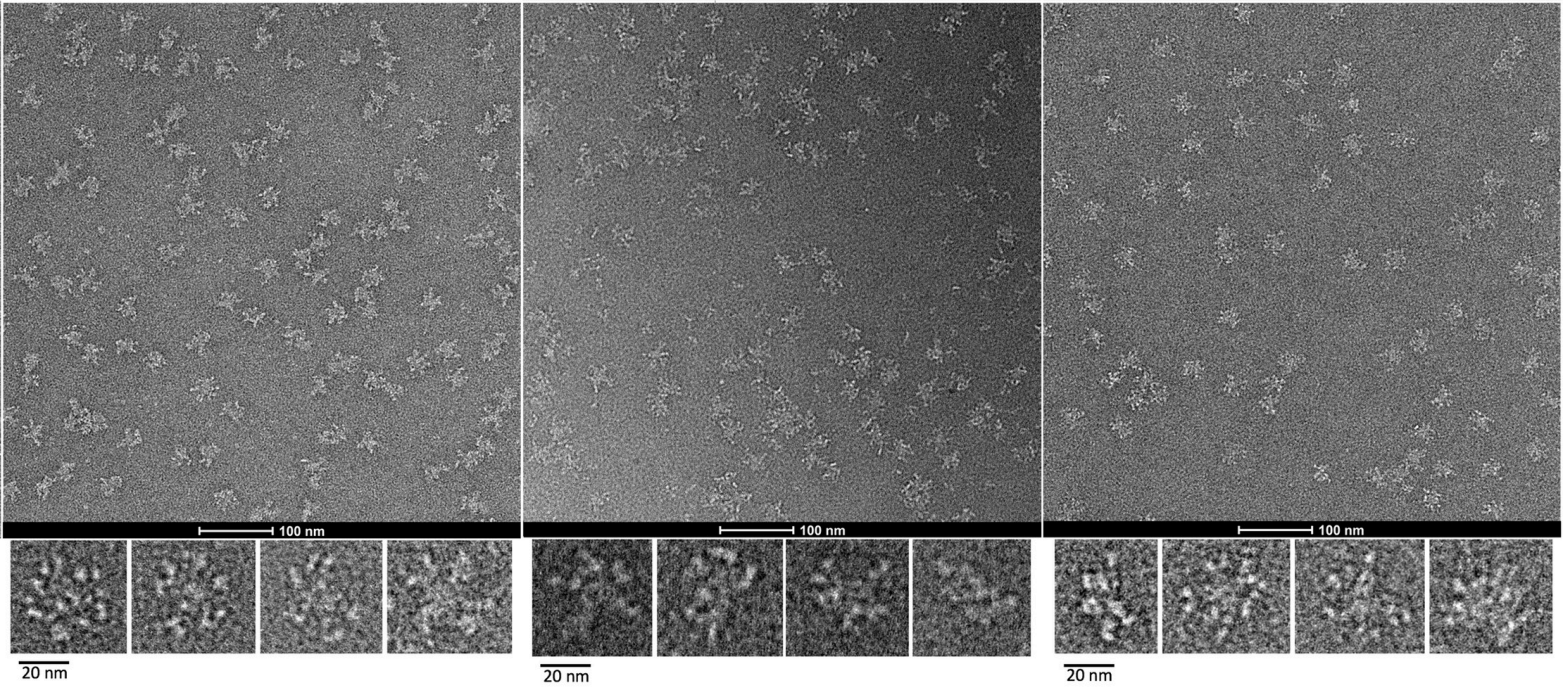
Non-processed images of negative stain transmission electron microscopy images of IgM antibody. Representative fields of particles with a 100 nm scale bar in the upper pictures. Magnified views of some individual molecules are shown in the lower panels for all three model antibodies produced in HEK293E cells: (A) IgM012, (B) IgM012_GL, (C) IgM617.

The structures of IgMs produced by HEK293E were analyzed with negative stain TEM in order to evaluate their structural integrity. The pentameric IgMs produced by HEK293E exhibited a central circular core with projecting Fab units in a star-shaped manner (Fig 2) as it is known for IgMs isolated from human serum [24,25]. A high degree of flexibility of the Fab units was observed as the Fab-domains were only rarely visible and, in some cases, less than five Fab arms were detected. This flexibility leads to a variety of possible conformations that are displayed by the enlarged isolated molecules in Fig 2. Symmetric and asymmetric shapes were found. Moreover, Fig 2 demonstrates that regions around Cμ2- and Cμ3-domains exhibit flexibility. A diameter of approximately 30–40 nm was found for pentameric IgM, which was already described in Czajkowsky and Shao, 2009 or Akhouri *et al*., 2016 [25,26]. Hence, IgMs transiently produced by HEK293E show similar structural properties as described for human serum derived IgM and as found for CHO produced IgM (S3 Fig). All three model antibodies showed IgM in its pentameric form (Fig 2), whereas a few dimeric forms of IgM012 and IgM012_GL might be visible (S3 Fig). Pentameric IgM with the appearance of the conventional model and only rarely aggregates could be found in all preparations.

### Antigen response of IgM produced in CHO or HEK cells

During recombinant production, it is of particular importance that the product exhibits its natural properties. In the case of antibodies, the interaction with the antigen is a key function. IgM617 and IgM012_GL were chosen as a representative to study the impact of the host cell line and expression system. The binding affinity of IgM617 to its natural antigens, glycosphingolipids, was evaluated by a ganglioside ELISA. Products of both expression hosts showed comparable binding to the gangliosides GM1, GD3 and GM3 (S4 Fig). Moreover, binding of IgM012_GL to its antigen UG37 was similar for CHO and HEK produced IgM (S5 Fig). These results indicate that the expression system, and the resulting biochemical characteristics as glycosylation pattern shown in Tables 1 and 2, do not affect the binding properties of IgM617 and IgM012_GL.

### Complement activation via C1q-IgM interaction

**Fig 3:**
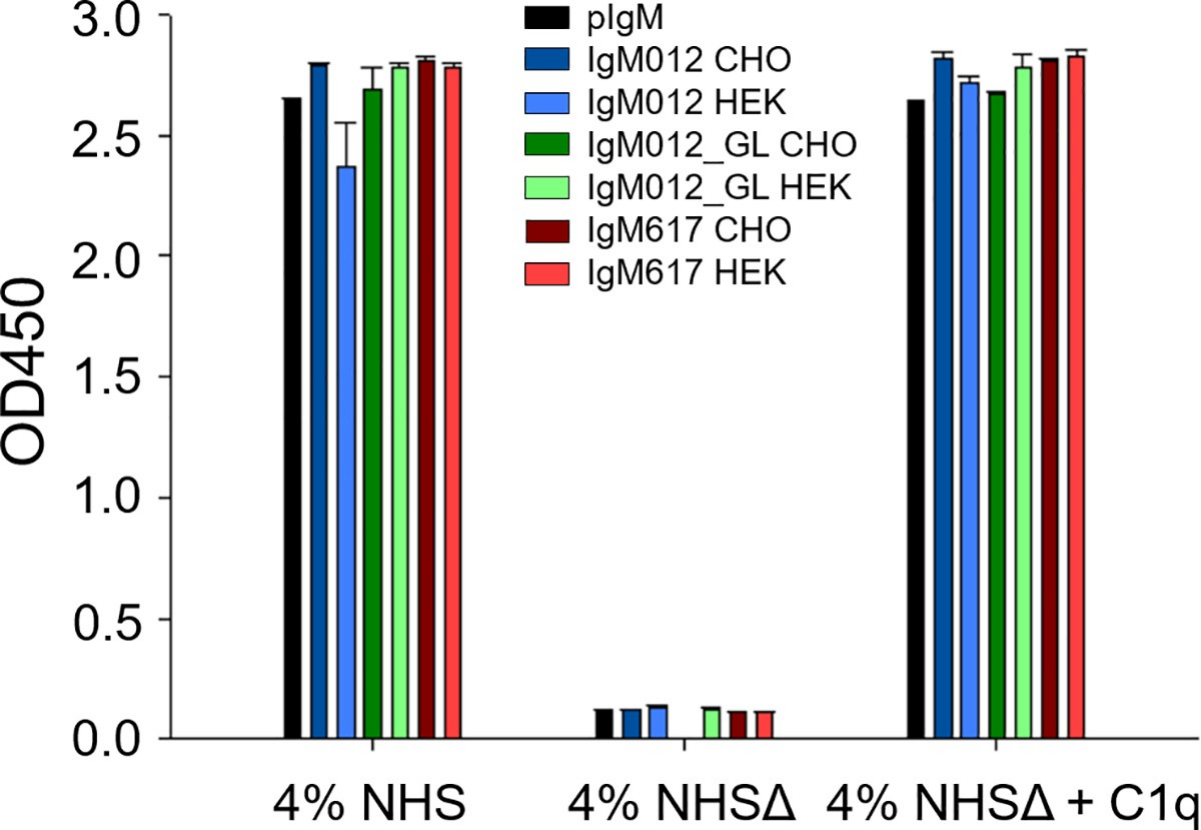
Complement activation via IgM and C1q interaction. Indicated IgMs were coated on the plate and incubated with normal human serum (NHS), NHS depleted of C1q (NHSΔ) or NHSΔ reconstituted with C1q. Polyclonal IgM (pIgM, black) as well as IgM012 (blue), IgM012_GL (green) and IgM617 (red) produced stably in CHO DG44 (darker colors) or transiently in HEK293E cells (lighter colors). Serum C4 cleavage by the IgM-bound activated C1 complex results in C4b deposition detected by a C4b specific antibody. All samples were analysed in duplicates.

The capacity of the different recombinant IgM preparations and polyclonal IgM isolated from human serum (pIgM) to trigger complement activation was analyzed and compared by ELISA as described in Bally *et al*., 2019 [20]. The assay is based on the detection of C4b fragment deposition after serum cleavage of C4 by the C1 complex bound to coated IgM molecules, which is a signature for the classical complement pathway activation. Assays with C1q-depleted normal human serum (NHSΔ) and C1q-reconstituted serum (NHSΔ + 4 μg/ml C1q) were used as controls for the C1q/IgM interaction dependency; pIgM was used as a reference. No significant differences were observed between pIgM and all the recombinant IgM samples (Fig 3), demonstrating that immobilized recombinant IgMs are as active as serum derived IgMs in these assay conditions, regardless their differences in polymer distributions and glycosylation patterns which would be a result either of their sequence or the host cell.

Indeed, although IgM variants, IgM012 and IgM012_GL, present 10 to 20% of dimers (Fig 1), they did not show any decrease in *in vitro* complement activation level (Fig 3). Taylor *et al*., 1994 demonstrated that IgM monomers are not able to provoke the activation of complement-mediated cytolysis [27]. Thus, reduction of complement activation by IgM mixture, as IgM012 and IgM012_GL was expected. Explanation might reside in the coated IgM dimer ability to activate the cascade or in the high sensitivity of the ELISA assay, since a few coated pentameric IgM molecules to the microtiter plate can be sufficient to activate the complement amplification cascade. Furthermore, and surprisingly, the oligomannose changes at GS4 between CHO DG44 and HEK293E production (Table 2), although minor, do not affect the activation level (Fig 3). It has been reported that the GS4 (Asn402) of IgMs may be important for the C1q binding and thus, complement-dependent cytolysis [7,28]. The similar extent of *in vitro* complement activation which we observed indicated that the presence of the oligomannose types at GS4 is more important for the complement activation than the exact structure or glycan composition.

## Discussion

Production of IgM antibodies constitutes a challenging task and new recombinant production strategies for development of new therapeutics and their evaluation are desperately needed. In this study, we present the successful transient expression of IgM antibodies in HEK cells and compared it to the traditional and well-established stable CHO cell system. It was shown that both systems enable comparable specific productivities and quality attributes, demonstrating that the transient expression in HEK cells represents a valuable alternative.

Firstly, all three model antibodies showed comparable specific productivities in the two cell systems (Fig 1). As previously described in Chromikova *et al*., 2015, tremendous differences in antibody titers between the individual IgM variants were observed [1]. Especially, production of IgM012 and IgM012_GL challenged both cell lines more than IgM617 production. Indeed, IgM012 and IgM012_GL expression resulted in lower IgM concentrations and more dimer fractions than IgM617 expression as well as an impaired glycosylation. Additionally, structural analysis using negative stain TEM and *in vitro* complement activation assays were used as new quality control attributes and confirmed the structural integrity and the functionality of all of our IgM models produced in both mammalian systems. Indeed, as described in previous studies [25,26], the conventional star-shaped particles of IgM pentamers was observed for all model IgMs produced in HEK cells (Fig 2 and S3 Fig).

Glycosylation is one of the most important quality attributes, as therapeutic application requires complex mammalian glycoforms since they are decisive for the function, folding and half-life time of protein products. Possible explanations for the differences in glycan structures might be environmental conditions, the host cell line or stress in course of the transient transfection mode. While environmental conditions are known to influence glycosylation of glycoproteins [29], this aspect can be neglected in case of complex IgM antibodies [22]. Although the expression systems led to different glycan patterns of the model IgMs, the potency of IgM617 to the glycosphingolipids GM1, GD3 and GM3 as well as the response of IgM012_GL to its antigen UG37 were not affected (S4 and S5 Figs). It is broadly accepted that glycosylation is dependent on the host cell line [29]. Although CHO and HEK cells are both widely used mammalian host cell lines, they exhibit variation in the complexity of their glycan patterns [30]. Unexpectedly, the HEK293E cell line produced a higher portion of truncated glycan structures with terminal N-acetylglucosamine, which is a less processed glycan pattern, compared to the CHO DG44 cell line, since a human cell line was supposed by its nature to enable human-like glycan structures (Table 1). Furthermore, the Man8 glycoform was significantly more frequent in GS4 of HEK293E derived IgM compared to the oligomannose structures found on GS4 of CHO DG44 derived IgM (Table 2). These differences could result from different expression levels of enzymes participating in glycosylation in the two host cell lines [31,32]. In accordance with Vestrheim *et al*., 2013, a lower sialic acid content was observed in HEK-derived immunoglobulins [33]. This is highly relevant when it comes to therapeutic usage, as sialic acid-rich proteins exhibit an extended half-life compared to non-sialylated proteins [34]. *In vitro* complement activation assay did not show any observable differences between pIgM and the recombinant IgMs, showing that recombinant IgM may be as active as serum derived IgM (Fig 3). Additionally, the transiently expressed IgM activated the complement system as efficiently as the stably expressed IgM. This might be due to the oligomannosidic carbohydrate structure at GS4 (Asn402) which is important for the C1q binding and complement-dependent cytolysis [7,35]. In contrast to previous observations [7,33,35], we did not observe differences in effective C1q binding due to glycosylation variations. However, sensitivity of the assay may not allow determining minor differences of activation, as an active fraction of pentamers may be sufficient to provoke a high signal and therefore complement activation did not reveal expression host dependency. Transient transfection poses an attractive method for fast and efficient IgM screening and a useful tool for fast IgM production. However, low reproducibility in transient transfections might contribute to high variabilities during transfections as a result of variabilities in transfectability of the host cell line [36]. Transient transfection may be accompanied by stress due to polyethyleneimine treatment and therefore result in less processed glycan forms. Intracellular glycan-processing machinery might suffer from the sudden switch to intensive protein production. Besides, product-associated variations in posttranslational modification were observed for several transiently produced proteins [30].

## Conclusions

In this study, the HEK293E transient expression system is able to produce complex multimeric proteins like IgMs and generates acceptable amounts and comparable polymer distribution as the well-established CHO produced IgM. However, the glycan profile was less processed, which could change the biological properties or pharmacokinetics [37]. Therefore, the transient expression in HEK293E should only be used for applications in which the glycan profile is not of specific interest or minor changes in the glycan profile have no impact. Nevertheless, the potency for complement activation was maintained and structural integrity demonstrated. Transient expression systems pose an attractive alternative for rapid, efficient and high-throughput production of complex IgM antibodies with possibly impaired post-translational modifications but proper effector properties.

## Supporting information

S1 Fig Schematic plasmid maps of the genetic constructs. (A) pIRES plasmids for stable transfection into CHO DG44 host cell line. Image was reprinted from Chromikova et al. [1]. (B) pCEP4 plasmids for transient transfection into HEK293E host cell line.
(PDF)

S2 Fig Immunoblotting of IgM012, IgM012_GL and IgM617 produced in CHO DG44 and HEK293E. Silver staining and western blots under reducing conditions were done for anti-*μ* and anti-κ chain.
(PDF)

S3 Fig Non-processed images of negative stain transmission electron microscopy images of IgM012 produced in CHO DG 44. Representative field of particles with a 200 nm scale bar. Circled particles represent side views of pentameric IgM012. The smaller particles pointed out by the yellow arrows indicate molecules that could be dimers.
(PDF)

S4 Fig Ganglioside-ELISA of GM1, GD3 and GM3 with IgM617 produced in CHO DG44 and HEK293E cells. Potency was tested with anti-hu IgM (μ-chain specific) alkaline phosphatase according to Zeng *et al*., 2005 [38].
(PDF)

S5 Fig Antigen (UG37) binding of IgM012_GL produced in CHO DG44 (green) and HEK293E cells (red). A microtiter plate was coated with UG37, washed and incubated with 50 *μ*L/well of IgM sample. Binding of the IgM to UG37 was detected with anti-kappa-HRP conjugate and TMB. All samples were analyzed in duplicates, except for the negative control, which was the IgM617 (black lines).
(PDF)

S1 Raw images (PDF)
