# Supplementary material for: Transient pentameric IgM fulfill biological function—Effect of expression host and transfection on IgM properties
Source: PLoS One. 2020 Mar 12;15(3):e0229992. doi: 10.1371/journal.pone.0229992 (PMC7067452; doi:10.1371/journal.pone.0229992)
Supplement: S1 Fig — (A) pIRES plasmids for stable transfection into CHO DG44 host cell line. Image was reprinted from Chromikova et al. [1]. (B) pCEP4 plasmids for transient transfection into HEK293E host cell line. (PDF) [file pone.0229992.s001.pdf]

Genetic constructs for stable and transient expression

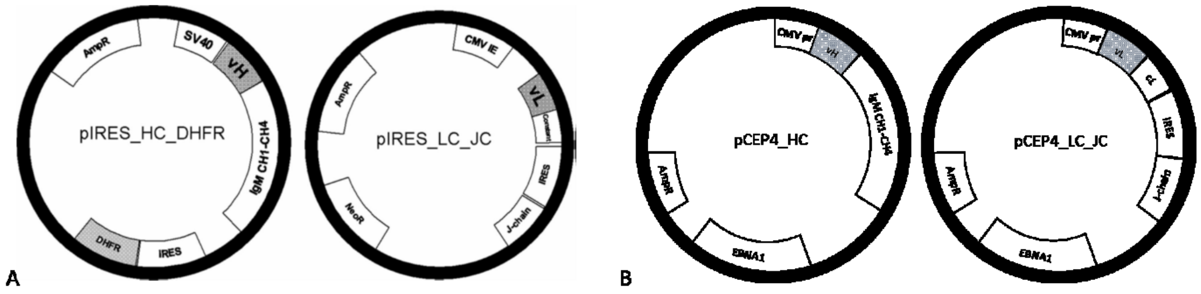

S1 Fig. Schematic plasmid maps of the genetic constructs. (A) pIRES plasmids for stable transfection into CHO DG44 host cell line. Image was reprinted from Chromikova et al. [16]. (B) pCEP4 plasmids for transient transfection into HEK293E host cell line.

Abbreviations: AmpR- ampicillin-resistant gene, SV40 – SV40 promoter, CMV – CMV promoter, vH/vL – variable domain of heavy chain/ light chain, IgM CH1-CH4- IgM constant domains, cL – kappa light chain constant domain, IRES - internal ribosome entry site, DHFR - dihydrofolate reductase, EBNA1 - Epstein-Barr virus nuclear antigen 1.
