# Supplementary material for: Transient pentameric IgM fulfill biological function—Effect of expression host and transfection on IgM properties
Source: PLoS One. 2020 Mar 12;15(3):e0229992. doi: 10.1371/journal.pone.0229992 (PMC7067452; doi:10.1371/journal.pone.0229992)
Supplement: S2 Fig — Silver staining and western blots under reducing conditions were done for anti-μ and anti-κ chain. (PDF) [file pone.0229992.s002.pdf]

Immunoblotting of IgM antibodies produced in CHO DG44 and HEK293E cells

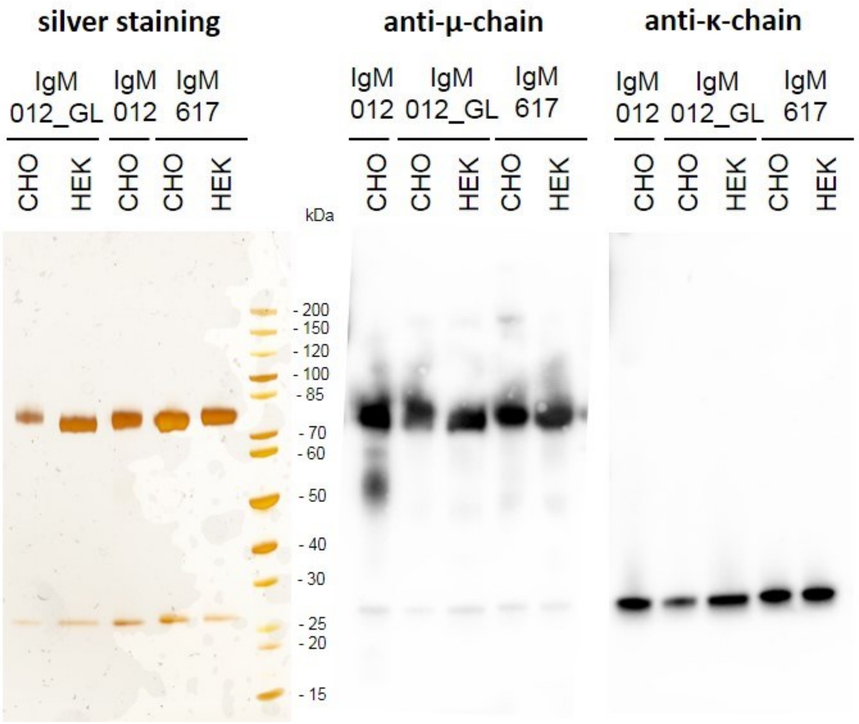

S2 Fig. Immunoblotting of IgM012, IgM012_GL and IgM617 produced in CHO DG44 and HEK293E. Silver staining and western blots under reducing conditions were done for anti-µ and anti-κ chain.
