# Supplementary material for: Transient pentameric IgM fulfill biological function—Effect of expression host and transfection on IgM properties
Source: PLoS One. 2020 Mar 12;15(3):e0229992. doi: 10.1371/journal.pone.0229992 (PMC7067452; doi:10.1371/journal.pone.0229992)
Supplement: S3 Fig — Representative field of particles with a 200 nm scale bar. Circled particles represent side views of pentameric IgM012. The smaller particles pointed out by the yellow arrows indicate molecules that could be dimers. (PDF) [file pone.0229992.s003.pdf]

Structural Analysis of pentameric and dimeric IgM012 produced by CHO DG44

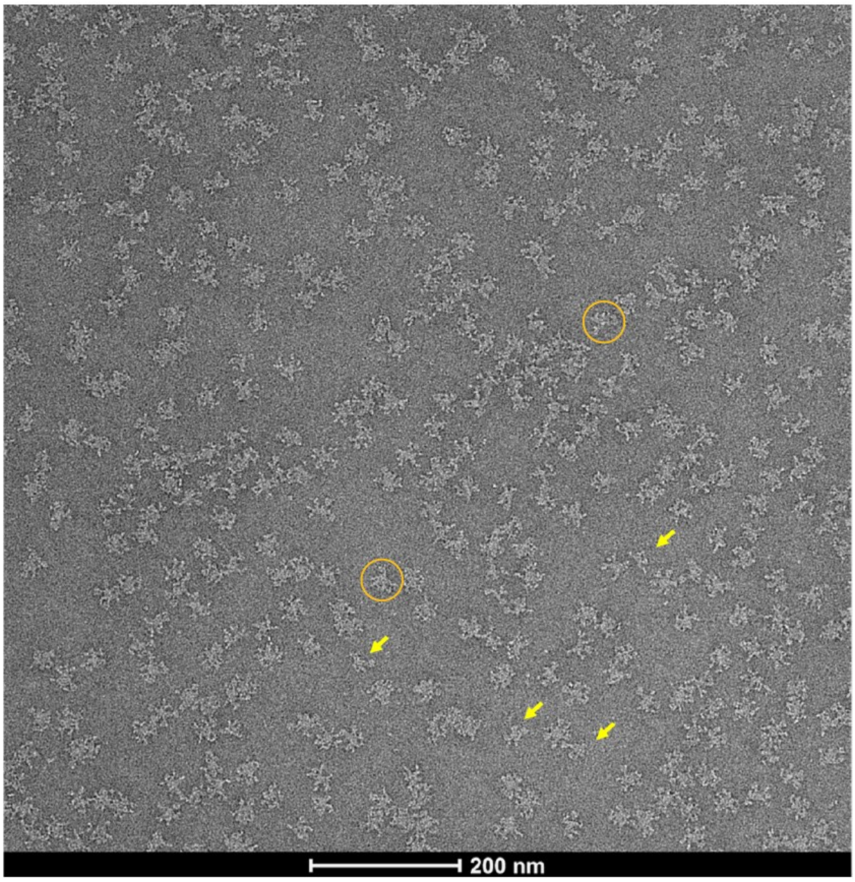

S3 Fig. Non-processed images of negative stain transmission electron microscopy images of IgM012 produced in CHO DG44. Representative field of particles with a 200 nm scale bar. Circled particles represent side views of pentameric IgM012. The smaller particles pointed out by the yellow arrows indicate molecules that could be dimers.
