# Supplementary material for: Transient pentameric IgM fulfill biological function—Effect of expression host and transfection on IgM properties
Source: PLoS One. 2020 Mar 12;15(3):e0229992. doi: 10.1371/journal.pone.0229992 (PMC7067452; doi:10.1371/journal.pone.0229992)
Supplement: S4 Fig — Potency was tested with anti-hu IgM (μ-chain specific) alkaline phosphatase according to Zeng et al., 2005 [38]. (PDF) [file pone.0229992.s004.pdf]

Antigen response

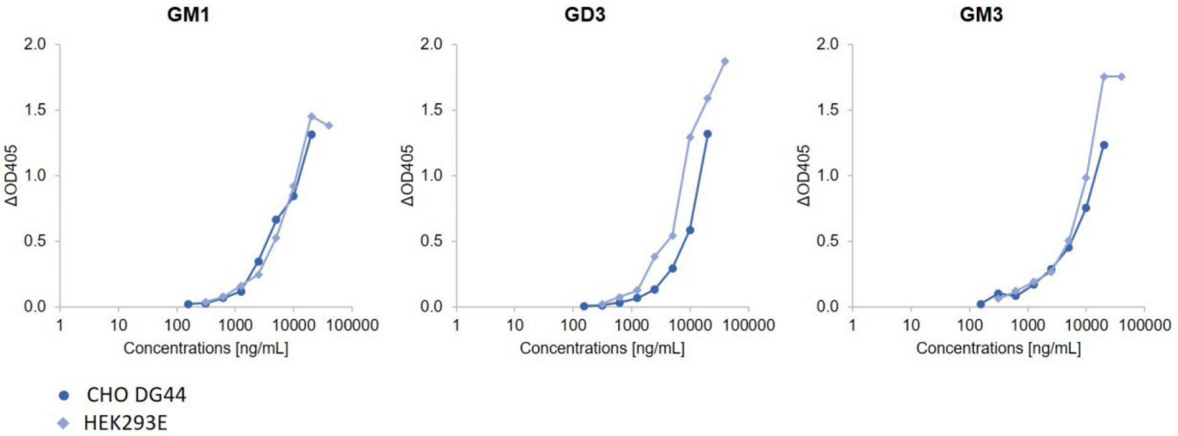

S4 Fig. Ganglioside-ELISA of GM1, GD3 and GM3 with IgM617 produced in CHO DG44 and HEK293E cells. Potency was tested with anti-hu IgM (μ-chain specific) alkaline phosphatase according to Zeng *et al.*, 2005.
