# Supplementary material for: Transient pentameric IgM fulfill biological function—Effect of expression host and transfection on IgM properties
Source: PLoS One. 2020 Mar 12;15(3):e0229992. doi: 10.1371/journal.pone.0229992 (PMC7067452; doi:10.1371/journal.pone.0229992)
Supplement: S5 Fig — A microtiter plate was coated with UG37, washed and incubated with 50 μL/well of IgM sample. Binding of the IgM to UG37 was detected with anti-kappa-HRP conjugate and TMB. All samples were analyzed in duplicates, except for the negative control, which was the IgM617 (black lines). (PDF) [file pone.0229992.s005.pdf]

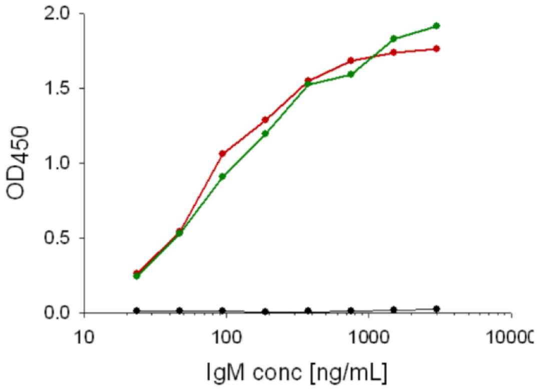

S5 Fig. Antigen (UG37) binding of IgM012_GL produced in CHO DG44 (green) and HEK293E cells (red). A microtiter plate was coated with UG37, washed and incubated with 50 µL/well of IgM sample. Binding of the IgM to UG37 was detected with anti-kappa-HRP conjugate and TMB. All samples were analyzed in duplicates, except for the negative control, which was the IgM617 (black lines).
