# Supplementary material for: Transient pentameric IgM fulfill biological function—Effect of expression host and transfection on IgM properties
Source: PLoS One. 2020 Mar 12;15(3):e0229992. doi: 10.1371/journal.pone.0229992 (PMC7067452; doi:10.1371/journal.pone.0229992)
Supplement: S1 Raw images — (PDF) [file pone.0229992.s006.pdf]

Original images for blots and gels

"Transient pentameric IgM fulfill biological function - effect of expression host and transfection on IgM properties"

Julia Hennicke[1], Linda Schwaigerlehner[1], Clemens Grünwald-Gruber[2], Isabelle Bally[3], Wai Li Ling[3], Nicole Thielens[3], Jean-Baptiste Reiser[3], Renate Kunert[1*]

[1] Department of Biotechnology, University of Natural Resources and Life Sciences, Vienna, Austria

[2] Department of Chemistry, University of Natural Resources and Life Sciences, Vienna, Austria

[3] Institut de Biologie Structurale, UMR 5075, Université Grenoble Alpes, CEA, CNRS, IBS, Grenoble, France

[*] Corresponding author

E-mail: renate.kunert@boku.ac.at (RK)

Contents

Original gel image of Fig 1 2

Original blot and gel images of S2 Fig 3

Original gel image of Fig 1.

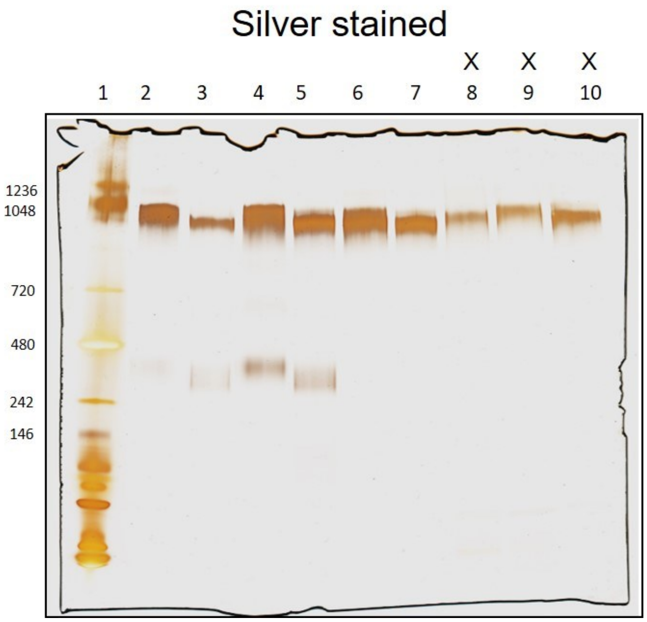

1 - Unstained Protein Marker	IgM-conc.
2 - IgM012 CHO	500 ng
3 - IgM012 HEK	200 ng
4 - IgM012_GL CHO	500 ng
5 - IgM012_GL HEK	500 ng
6 - IgM617 CHO	500 ng
7 - IgM617 HEK	500 ng
8 - IgM103	500 ng
9 - IgM104	500 ng
10 - IgM617	500 ng

Image capturing by Epson Perfection V800 Photo

X not included in the final figure

Fig 1. Original image of gel for polymer distribution of IgM antibodies produced in CHO DG44 and HEK293E cells. Silver staining under non-reducing conditions of purified IgM012, IgM012_GL and IgM617 (and IgM103, IgM104 and IgM617) produced stably in CHO cells and transiently in HEK cells. All lanes, which are excluded in Fig 1, are indicated with an "X".

Original blot and gel images of S2 Fig

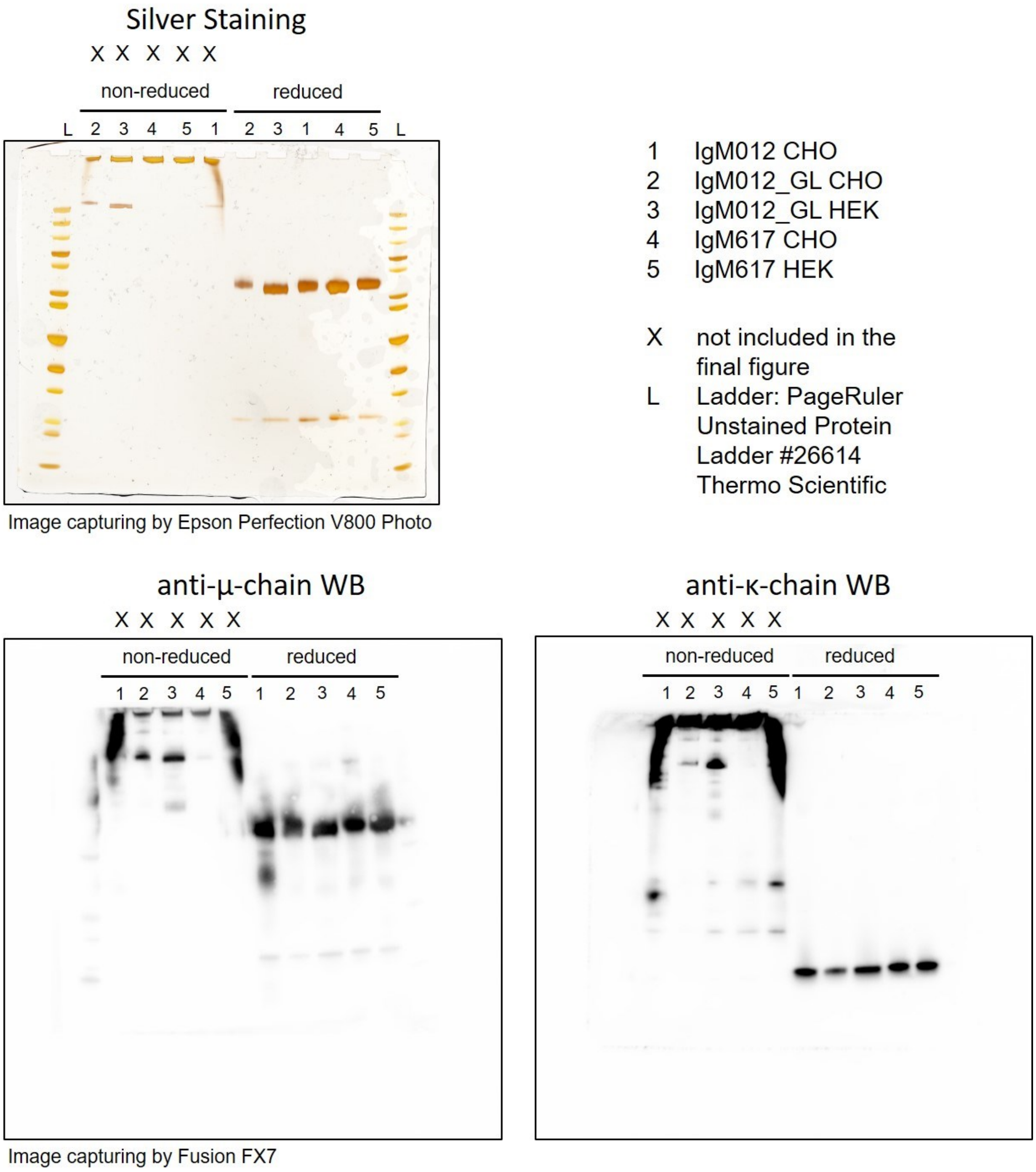

S2 Fig. Original images for blots and gel. Immunoblotting of IgM012, IgM012_GL and IgM617 produced in CHO DG44 and HEK293E. Silver staining and western blots under reducing conditions were done for anti-µ and anti-κ chain. All lanes, which are excluded in S2 Fig, are indicated with an “X”.
